# Identifying healthcare transmission routes of nontuberculous mycobacteria with whole genome sequencing: a systematic review

**DOI:** 10.1017/ice.2025.6

**Published:** 2025-04

**Authors:** Spencer D. Schrank, Dale Z. Gozum, Elise M. Martin, Graham M. Snyder

**Affiliations:** 1 Division of Infectious Diseases, University of Pittsburgh School of Medicine, Pittsburgh, PA, USA; 2 Department of Infection Prevention and Control, UPMC Presbyterian/Shadyside, Pittsburgh, PA, USA; 3 Department of Medicine, UPMC Lititz, Lititz, PA, USA; 4 Veterans’ Affairs Pittsburgh Healthcare System, Pittsburgh, PA, USA

## Abstract

**Objective:**

To enumerate and describe the effect of whole genome sequencing (WGS) on epidemiological investigations of healthcare-associated transmission of nontuberculous mycobacteria (NTM).

**Design:**

Systematic review.

**Methods:**

We performed a literature search using targeted search terms to identify articles meeting inclusion criteria. Data extraction of study characteristics and outcomes was performed by two independent researchers. The primary outcome was the author interpretation of WGS utility in the investigation of suspected healthcare-associated transmission of NTM. The secondary outcome was whether a transmission route was identified through WGS.

**Results:**

Thirty-one studies were included in the final analysis with 28 (90%) concluding that WGS was helpful in transmission investigations and in 19 of these 28 (68%) WGS aided in identifying a transmission route. The most common identified transmission routes were water-borne point sources (10), heater-cooler units (6), patient-to-patient (4), and a healthcare worker (1).

**Conclusion:**

WGS is an informative tool in investigating healthcare transmission of NTM.

## Introduction

Nontuberculous mycobacteria (NTM) are ubiquitous environmental organisms that have been attributed to healthcare-associated transmission events for both local and global outbreaks.^1^ Traditional epidemiological investigation methods of NTM healthcare transmission require identification of an outbreak by clinicians, infection prevention teams or microbiologists. Prolonged incubation periods, limited microbiologic techniques, and varying clinical presentations can make case ascertainment and identification of transmission pathways difficult.^2^

Whole genome sequencing (WGS) has become the gold standard for epigenomic investigations of healthcare-associated transmission.^3^ WGS has been most often used as a reactive tool to supplement traditional epidemiologic investigations of suspected healthcare-associated outbreaks but has more recently been used as a mode of surveillance to identify healthcare transmission when an outbreak is not apparent.^4^ WGS to identify healthcare transmission routes of NTM is becoming more prevalent, but additional studies are required to understand its utility supplementing traditional epidemiological methods.

In this systematic review, we aim to enumerate and describe the studies that have used WGS to identify or characterize healthcare-associated transmission of NTM. The purpose of this review is to characterize the reported value of adding WGS to traditional epidemiological investigation methods in identifying transmission routes, and therefore, opportunities to prevent nosocomial transmission of NTM. As WGS becomes more available and routinely used for pathogen transmission investigation, infection prevention teams can allocate resources to prevent transmission in healthcare settings.

## Methods

### Study selection

**Figure 1.**
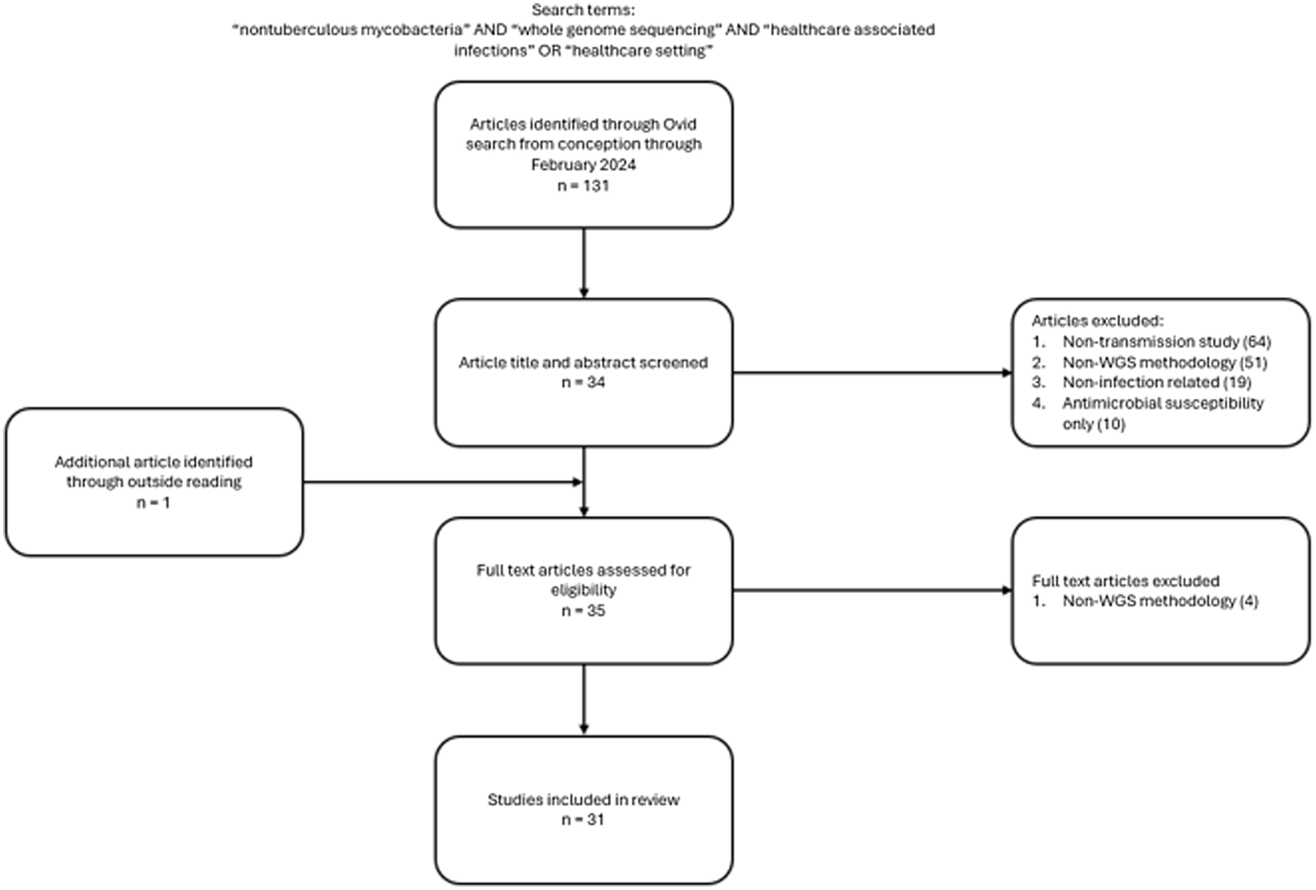
Flow diagram of study selection.

A literature search was performed In Ovid MEDLINE ALL from inception to February 2024 (Figure 1). The search terms “nontuberculous mycobacteria” AND “whole genome sequencing” AND (“healthcare-associated infections” OR “healthcare setting”) were used. Results were restricted to English language. The title and abstract were screened for eligibility to exclude studies that were non-infection related, only antimicrobial susceptibility related, or selectively used non-WGS methods for transmission investigation. The Preferred Reporting Items for Systematic Reviews and Meta-Analyses (PRISMA) guidelines were followed for reporting the results of this study.^5^

Data extraction was performed in duplicate by two independent physician researchers (SS, DG) with any discordances resolved through a joint evaluation. These variables included study, patient, infection, and genomic characteristics of each study (Supplemental Table 1).

### Data analysis

The primary outcome was the study authors’ conclusion about WGS utility in establishing or refuting a healthcare-associated transmission route. A secondary outcome included whether WGS identified a specific transmission route.

Qualitative characteristics were summarized by the frequency with which they occur. The following study characteristics were summarized by measures of center: study duration, case and control patient and isolate frequencies, and SNP cutoff (if reported).

Microsoft Forms was used for data extraction for each study by both independent reviewers. Results were imported into Microsoft Excel after data extraction was completed by both reviewers. Qualitative and quantitative summary statistics were performed in Microsoft Excel.

Ethical and patient consent was not required for this study because data was retrieved and synthesized from previously published studies.

## Results

One-hundred and thirty-one articles were identified using the initial search strategy. After screening all articles by titles and abstracts, 35 (27%) articles met screening criteria. One additional article was identified through a cited reference. Thirty-one articles were included in the final analysis after full text review (Figure 1).^6–36^

Most studies were cohort design (23, 74%). Thirty-nine percent (12) were acute-care hospitals, 26% (8) were multi-facility, 23% (7) were mixed inpatient and outpatient facilities, 10% (3) were outpatient clinics, and 3% (1) was a long-term care facility. The median study duration was 5 years (interquartile range [IQR], 6) (Table 1). The most frequently investigated organisms were *Mycobacteroides abscessus* (16 studies totaling 3,681 isolates), *Mycobacterium chimaera* (8 studies, 111 isolates), and *Mycobacterium fortuitum* complex (2 studies, 42 isolates); 11 other species were reported across 9 studies, totaling 147 isolates. The median number of patient-cases investigated per study was 22 (IQR, 45) with 25 (IQR, 78) patient-isolates. For studies that included control cases (n = 6), the median number of control patients per study was 15 (IQR, 13) with 10 (IQR, 14) patient-isolates. In studies that reported anatomic site of isolate collection, a total of 3,549 isolates were described with the most common anatomical site of collection being the lungs (n = 3,174 isolates, 89%) (Supplemental Table 2). The most frequently reported HAI was surgical-site infection (n = 333 isolates, 87%) (Supplemental Table 3) with the lungs being the most sequenced anatomic site of collection among studies that reported the breakdown of sequenced isolates implicated in transmission by anatomic site (n = 762 isolates, 74%) (Supplemental Table 4).

**Table 1.** Characteristics of studies reporting the use of whole genome sequencing to investigate potential transmission of non-tuberculous mycobacteria

					Cases		Controls		
Publication First Author, Year	Study Design	Study Duration (Years)	Facility Type	NTM Species Investigated	Patients	Isolates	Patients	Isolates	SNP Cutoff
Bryant, 2013^6^	Cohort	4	MIO	*Mycobacteroides abscessus* complex	31	168			50
Harris, 2015^8^	Cohort	NR	MIO	*Mycobacteroides abscessus* complex	20	27			25
Robinson, 2016^9^	Case Report	1	MF	*Mycobacterium chimaera*	1	1	1	1	NR
Chand, 2016^10^	Case-Control	8	MF	*Mycobacterium chimaera*	15	29	159	200	NR
Everall, 2017^11^	Cohort	6	MF	*Mycobacteroides abscessus* complex	14	14			NR
Hasan, 2019^12^	Case-Control	2	MF	*Mycobacterium chimaera*	24	24	7	8	NR
Decalonne, 2019^13^	Case Report	1	OC	*Mycobacteroides chelonae*	1	1			NR
Gu, 2020^14^	Cohort	1	ACH	*Mycobacteroides chelonae*	7	7			NR
Yan, 2020^7^	Cohort	4.3	MIO	*Mycobacteroides abscessus* complex	22	22			20
Labuda, 2020^15^	Cohort	0.75	OC	Unknown species	52	52			NR
Doyle, 2020^16^	Cohort	16	MIO	*Mycobacteroides abscessus* complex	62	145			NR
Ghodousi, 2020^17^	Cohort	5	ACH	*Mycobacterium chimaera*	23	23	15	15	NR
Wetzstein, 2020^18^	Cohort	12	MIO	*Mycobacteroides abscessus* complex	29	39			25
Xu, 2021^19^	Cohort	2.58	ACH	*Mycobacterium chimaera*	18	25			NR
Lipworth, 2021^20^	Cohort	4.83	MIO	*Mycobacteroides abscessus* complex	906	2431			25
Lecorche, 2021^21^	Cohort	6.83	ACH	*Mycobacterium chimaera*	2	3			NR
Fujiwara, 2022^23^	Cohort	NR	ACH	*Mycobacteroides abscessus* complex	24	25			25
Waglechner, 2022^24^	Cohort	5	OC	*Mycobacteroides abscessus* complex	7	288	7	10	25
Daniau, 2022^25^	Cohort Cohort Cohort Cohort Cohort Cohort Cohort Cohort Cohort Cohort Cohort	7.5 7.5 7.5 7.5 7.5 7.5 7.5 7.5 7.5 7.5 7.5	MF MF MF MF MF MF MF MF MF MF MF	*Mycobacterium fortuitum* complex *Mycobacteroides chelonae* *Mycobacteroides abscessus complex* *Mycobacterium mucogenicum* *Mycobacterium chimaera* *Mycobacterium avium* complex *Mycobacterium neoaurum* *Mycobacterium lentiflavum* *Mycobacterium marinum* *Mycobacterium fuerthensis* *Mycobacterium wolinskyi*		42 30 14 5 4 2 2 1 1 1 1			10 10 10 10 10 10 10 10 10 10 10
Inkster, 2022^26^	Cohort	1	ACH	*Mycobacteroides chelonae*	2	2			NR
Davidson, 2022^27^	Cohort	1.4	ACH	*Mycobacteroides abscessus* complex	7	7	15	15	NR
Gross, 2022^22^	Cohort	7	MF	*Mycobacteroides abscessus* complex	27	27			10
Wetzstein, 2022^28^	Cohort	4	MF	*Mycobacteroides abscessus* complex	123	154			25
Dedrick, 2023^29^	Cohort	2	MF	*Mycobacteroides abscessus* complex	90	90			7
Gross, 2023^36^	Cohort Cohort	2 2	MIO MIO	*Mycobacterium avium* complex *Mycobacterium chimaera*	9 2	9 2			30 30
Nagano, 2023^30^	Cohort	1	ACH	*Mycobacterium lentiflavum*	22	22			NR
Komiya, 2023^31^	Cohort	4	LTCF	*Mycobacteroides abscessus* complex	52	52			NR
Faury, 2023^32^	Case Report	7.3	ACH	*Mycobacterium fortuitum*	1	1			NR
Kling, 2023^33^	Cohort	0.5	ACH	*Mycobacterium xenopi*	6	3			NR
Groenewald, 2023^34^	Case-Control Case-Control	1.5 1.5	ACH ACH	*Mycobacterium goodii* *Mycobacterium wolinskyi*	3 5	3 5			NR NR
Klompas, 2023^35^	Cohort	3	ACH	*Mycobacteroides abscessus* complex	4	4			40

Note. ACH, inpatient acute care hospital; LTCF, long-term care facility; MIO, mixed inpatient and outpatient; MF, multi-facility; NR, not reported; NTM, non-tuberculous mycobacteria; OC, outpatient clinic; SNP, single nucleotide polymorphism.

Among the 14 (45%) studies reporting SNP, the median SNP cutoff used for genetic relatedness was 25 (range, 5–30 SNPs) (Table 1). Eighteen (58%) studies used reactive WGS and 13 (42%) studies used WGS surveillance to identify a potential transmission route among epidemiologically linked infections. Twenty-eight (90%) of the studies in this review determined that WGS aided in their investigation of NTM healthcare transmission (Table 2, Supplemental Table 5). Of these 28 studies, 19 (68%) identified a transmission route using WGS. One (3%) study concluded that WGS did not provide additional information in their transmission investigation and two (6%) studies did not report whether WGS aided in their investigation, despite the authors suspecting patient-to-patient transmission routes. Transmission routes identified were water-borne (n = 10), associated with heater-cooler units (n = 6), patient-to-patient (n = 4) and related to a healthcare worker (n = 1). The water-borne transmission routes included both potable and non-potable water sources. Patient-to-patient transmission routes were identified among cystic fibrosis patients in two studies, chronically ventilated non-cystic fibrosis patients in one study and was unspecified in another study. One study identified multiple transmission routes. Environmental culture analysis, including WGS, was performed in 61% (19) of studies. (Table 2).

**Table 2.** Author-reported transmission routes and utility of whole genome sequencing in investigations of infection clusters attributable to non-tuberculous mycobacteria

Publication First Author, Year	Transmission route identified	Environmental culture analysis with WGS performed	Did sequencing aid in identifying point source of outbreak?	What type of WGS method was used?
Bryant, 2013^6^	Patient-to-patient	Yes	Yes	Active
Harris, 2015^8^	None	Not performed	Yes	Active
Robinson, 2016^9^	None	Yes	Yes	Reactive
Chand, 2016^10^	HCU	Yes	Yes	Reactive
Everall, 2017^11^	None	Not performed	Yes	Reactive
Hasan, 2019^12^	HCU	Yes	Yes	Reactive
Decalonne, 2019^13^	Clinic water supply	Yes	Yes	Reactive
Gu, 2020^14^	None	Not performed	Yes	Reactive
Yan, 2020^7^	Pulmonary function testing	Not performed	Yes	Active
Labuda, 2020^15^	Water flush	Yes	Yes	Reactive
Doyle, 2020^16^	None	Not performed	Yes	Active
Ghodousi, 2020^17^	HCU	Yes	Yes	Reactive
Wetzstein, 2020^18^	None	Not performed	Yes	Active
Xu, 2021^19^	HCU	Yes	Yes	Reactive
Lipworth, 2021^20^	None	Not performed	Yes	Active
Lecorche, 2021^21^	HCU	Yes	Yes	Reactive
Fujiwara, 2022^23^	Patient-to-patient	Not performed	Not reported	Active
Waglechner, 2022^24^	None	Not performed	No	Active
Daniau, 2022^25^	HCU Water faucet Autologous stem cell transplant	Yes	Yes	Reactive
Inkster, 2022^26^	Water faucet	Yes	Yes	Reactive
Davidson, 2022^27^	Facility water	Yes	Yes	Reactive
Gross, 2022^22^	Community water	Yes	Yes	Active
Wetzstein, 2022^28^	None	Not performed	Yes	Active
Dedrick, 2023^29^	Patient-to-patient	Not performed	Not reported	Active
Gross, 2023^36^	Drinking fountain	Yes	Yes	Active
Nagano, 2023^30^	Tap water	Yes	Yes	Reactive
Komiya, 2023^31^	Patient-to-patient	Yes	Yes	Active
Faury, 2023^32^	Shower head	Yes	Yes	Reactive
Kling, 2023^33^	None	Not performed	Yes	Reactive
Groenewold, 2023^34^	Healthcare worker	Yes	Yes	Reactive
Klompas, 2023^35^	Ice machine	Yes	Yes	Reactive

## Discussion

We identified 31 publications describing investigations of healthcare-associated infection due NTM using WGS to characterize the outbreak. Of these 31 studies, 28 (90%) described WGS as being helpful in their investigation and 19 (61%) studies identified a transmission route through WGS. Based on this published experience, WGS is a useful tool to aid investigations of clusters of NTM infections.

Traditional epidemiological investigations of NTM transmission are challenging because of the microbiologic characteristics of these organisms. Previous studies have supported WGS in identifying HAI transmission routes of various NTM pathogens, but prior to this review, there has been limited assessment of the effectiveness of WGS on NTM transmission route identification.^37^ Through WGS, most authors were able to identify or hypothesize transmission routes, with water-borne point sources including potable water, water flushes, and ice machines being most common, followed by heater-cooler-units and patient-to-patient transmission. A recent review by Abbas et al described both healthcare-associated and nonhealthcare-associated transmission of NTM. They concluded that hospital water systems act as a reservoir for NTM leading to equipment contamination with subsequent transmission.^38^ Identification of specific transmission routes allowed authors to perform targeted interventions within their facility or in the case of more regional studies, give insight into infection prevention and control practices.

WGS in transmission investigations can be used as both a reactive tool and an active surveillance tool.^39^ This review identified both methods being deployed by authors in their investigations (Table 2). For example, Davidson *et al.* performed WGS on *M. abscessus* isolates that were already implicated in a biphasic hospital outbreak and additionally performed WGS on environmental samples as part of their epigenomic investigation. Patient and environmental isolates were found to be highly genetically related, confirming their hypothesis that the hospital water system was the source of their original outbreak.^27^ Although their retrospective epigenomic investigation identified a transmission route, if active surveillance had been performed, disruption in transmission during their biphasic outbreak might have occurred sooner. Alternatively, Komiya et. al performed active surveillance sequencing of isolates to investigate whether there was undetected transmission of *M. abscessus* among ventilator dependent patients without cystic fibrosis. Their findings suggested patient-to-patient transmission.^31^ This contrast in surveillance methodology demonstrates the ability for WGS active surveillance to identify undetected transmission events.

The anatomical site of infection may also provide insight into the risk of prior healthcare-associated transmission. Omori et al found that 9% of their patients had extrapulmonary NTM infections. Of these patients the most prominent sites of infection were skin and soft tissue (44%) followed by blood (20%).^40^ In this systematic review we found that the anatomic site most often implicated in healthcare-associated transmission of NTM by WGS was skin and soft tissue (194, 53%). We can hypothesize that patients identified with extrapulmonary NTM infections with recent healthcare exposure may warrant additional investigation, including WGS, for potential healthcare-associated transmission.

This review also builds a collection of evidence that WGS is essential for HAI transmission investigations by supporting infection prevention teams in identifying transmission routes that were previously unrecognized. Kling *et al.* identified a doubling in their case rates of *Mycobacterium xenopi* respiratory cultures during a six-month period, finding no epidemiological links through their investigation to support an outbreak or pseudo-outbreak. WGS was performed which found the isolates to be genetically dissimilar, supporting the hypothesis that NTM acquisition was likely outside the healthcare setting. Harris *et al.* reviewed epidemiologic data of a pediatric cystic fibrosis cohort that showed limited spatiotemporal healthcare interaction among patients with *M. abscessus.* WGS performed on their isolates also showed no genetic relatedness among these patients, refuting potential cross-transmission in the healthcare setting.^33^ These studies show the importance of WGS as a tool to exclude healthcare transmission more particularly when epidemiological investigations do not identify transmission routes but cases show temporospatial clustering.

This study has several limitations. Publication bias likely exists within this review but given the descriptive nature of these studies assessing publication bias through statistical tools such as funnel plots is not feasible.^8^ Several studies were only able to ascertain patient data through large national health records, preventing investigators from identifying specific transmission routes. SNP threshold analysis has been studied for *M. abscessus* and *M. chimaera* given the prior experience with healthcare-associated outbreaks.^12,41,42^ SNP interpretations should continue to be done cautiously since variation does exist and there is limited data behind SNP thresholds of other species of NTM. This method of SNP analysis is commonly adopted but other methods of evaluating genetic relatedness may emerge (Supplemental Table 6). This review focused on the SNP analysis reported by the authors though the studies also described additional genomic analyses (Supplemental Table 7). Lastly, this review reports on the authors’ conclusions of transmission routes and utility of WGS but does not further identify the additional interventions to stop additional transmission or the incremental effectiveness of transmission interruption (and therefore, case prevention) that adding WGS to traditional epidemiological methods may afford. Although these interventions can be inferred, the details were not always reported and would be therefore difficult to validate.

Although studies have shown the effectiveness of WGS in healthcare transmission investigations, no prior studies have summarized whether it can be used effectively for NTM. This systematic review supports the need for including NTM as part of epidemiological investigations with WGS and traditional epidemiological investigations.

## Supporting information

Schrank et al. supplementary material Schrank et al. supplementary material

## Supplementary material

The supplementary material for this article can be found at https://doi.org/10.1017/ice.2025.6
