# Supplementary material for: Identifying healthcare transmission routes of nontuberculous mycobacteria with whole genome sequencing: a systematic review
Source: Infect Control Hosp Epidemiol. 2025 Feb 3;46(4):404–9. doi: 10.1017/ice.2025.6 (PMC12015626; doi:10.1017/ice.2025.6)
Supplement: Schrank et al. supplementary material [file S0899823X25000066sup001.pdf]

Supplemental Data

Identifying healthcare transmission routes of nontuberculous mycobacteria with whole genome sequencing: A systematic review. Schrank SD, et al.

Table of contents

Supplemental Table 1……2

Supplemental Table 2……3

Supplemental Table 3……6

Supplemental Table 4……7

Supplemental Table 5……8

Supplemental Table 6……11

Supplemental Table 7……12

Supplemental Table 1. Study variables collected

**Study Variable**
Study design
Study duration
Facility Type
Epidemiologic case definition
Control group definition (if applicable)
Patient frequency - cases
Patient frequency - controls
Species
Species-isolate frequency
Anatomical site of infection frequency
Healthcare-associated infection (HAI) type (as reported by author)
HAI frequency
Epidemiologic exposure
Single nucleotide polymorphism (SNP) cutoff
WGS as a reactive tool (add information to help interpret 94 epidemiological findings from an investigation of a previously defined cluster)
WGS as active surveillance tool (perform WGS on all isolates of a group prior to identifying a cluster based on epidemiological criteria)
Transmission route identification
Effectiveness of WGS in NTM transmission investigations (as interpreted by the author)

Supplemental Table 2. Anatomical sites of isolates

1st Author, Year	Anatomic Site	Frequency
**Bryant, 2013**	Respiratory	168
**Harris, 2015**	Respiratory	27
**Robinson, 2016**	Respiratory	1
**Meera, 2016**	Cardiac	11
	SST	3
	Disseminated	3
	Bone	1
**Everall, 2017**	SST	1
**Yan, 2020**	Respiratory	118
**Hasan, 2019**	NR	
**Decalonne, 2019**	SST	1
**Gu, 2020**	SST	4
	Respiratory	3
**Labuda, 2020**	Blood	52
**Doyle, 2020**	Respiratory	134
	Gastrointestinal	11
	Blood	1
**Ghodousi, 2020**	Blood	19
	Gastrointestinal	2
	CNS	1
	Respiratory	1
**Wetzstein, 2020**	Respiratory	38
	Lymph node	1
**Xu, 2021**	SST	1
	Cardiac	4
	Deep fluid collection	2
	Blood	1
	Bone	1

	Respiratory	1
	Stool	1
**Lipworth, 2021**	Respiratory	1997
	BAL	135
	Unknown	71
	Tissue Unclear Source	14
	Blood	19
	Cutaneous Tissue	10
	Pus	9
	Peritoneal dialysis catheter	8
	Swab	8
	Breast	4
	Aspirate unclear source	2
	Fluid unclear source	4
	Intraabdominal	4
	Pleural fluid	3
	Chin implant	2
	Lung tissue	2
	Lymph biopsy	1
	Spinal Aspirate	1
	Tibia aspirate	1
**Lecorche, 2021**	Blood	2
	Bone	1
**Gross, 2022**	Respiratory	27
**Fujiwara, 2022**	Respiratory	25
**Waglechner, 2022**	Respiratory	238
**Daniau, 2022**	SST	31
	Bone	15
	Blood	14
	Ocular	4

	Gastrointestinal	2
	Urogenital	1
**Inkster, 2022**	Blood	2
**Davidson, 2022**	Respiratory	16
	Pleural Fluid	2
	SST	2
	Intraabdominal	1
	Blood	1
**Gross, 2023**	Respiratory	11
**Nils, 2022**	Respiratory	154
**Dedrick, 2023**	Respiratory	NR
	SST	NR
**Nagano, 2023**	Respiratory	22
**Komiya, 2023**	Respiratory	52
**Faury, 2023**	Blood	1
**Kling, 2023**	Respiratory	6
**Groenewold, 2023**	Bone	8
**Klompas, 2023**	Blood	2
	SST	2

Note: SST, Skin/Soft Tissue Infection; NR, Not Reported; CNS, Central Nervous System; BAL, Bronchoalveolar Lavage

Supplemental Table 3. Frequency of author defined hospital-associated infections (HAI) type among non-tuberculous mycobacterial infections in cluster investigations aided by whole genome sequencing

Publication First Author, Year	Surgical Site Infection (% total infections)	Central Line Associated Blood Stream Infection (% total infections)	Hospital Acquired Pneumonia (% total infections)	Total study infections
**Chand, 2016**	18 (83%)			15
**Everall, 2017**	188 (100%)			188
**Decalonne, 2019**	1 (100%)			1
**Gu, 2020**	2 (29%)			7
**Labuda, 2020**	52 (100%)			52
**Xu, 2021**	12 (67%)		1 (6%)	18
**Daniau, 2022**	51 (60%)	17 (20%)		85
**Inkster, 2022**		1 (50%)		2
**Faury, 2023**		1 (100%)		1
**Groenewold, 2023**	7 (88%)			8
**Klompas, 2023**	2 (50%)			4
**Total**	333 (87%)	19 (5%)	1 (0.3%)	381

Note: Publications not listed did not explicitly document hospital-associated infections.

Supplemental Table 5. Details of authors' assessment of the importance of whole genome sequencing in investigations of clusters of infections due to non-tuberculous mycobacteria

Publication First Author, Year	Did sequencing aid in identifying point source of outbreak?	Author comments
Bryant, 2013	Yes	Whole genome sequencing has provided the first convincing evidence for transmission of *M. abscessus.*
Harris, 2015	Yes	In conclusion, we could not demonstrate cross-transmission of *M. abscessus* within our hospital, except between 1 sibling pair that had intense exposure both in the hospital and, perhaps more importantly, the home environment. Two patients were infected with highly genetically similar strains but appeared to be epidemiologically unrelated.
Robinson, 2016	Yes	Whole-genome sequencing (WGS) revealed the phylogenetic relationships, dominant circulating clones (DCCs), the likelihood of patient-to-patient transmission and the presence of prophages.
Chand, 2016	Yes	WGS analysis detected very low genetic diversity across probable cases, which were restricted to a single lineage within the M. chimaera species. The absence of any genetically divergent M. chimaera among probable cases and high genetic similarity to HCU isolates is consistent with a role for HCUs in transmission.
Everall, 2017	Yes	The clear geographical and temporal structure within the phylogeny, is a pattern more consistent with the introduction of the lineage to different locations occurring through transmission, rather than dissemination from a point source.
Yan, 2020	Yes	This cluster of isolates with a difference of <7 SNPs (i.e. equivalent to or less than same-patient isolate difference) suggests a shared recent ancestor and high probability of cross-transmission.
Hasan, 2019	Yes	The application of WGS has advanced our understanding of M. chimaera present in US LivaNova 3T HCUs and patient cases after the initial analysis of suspected cases in Pennsylvania and Iowa.
Decalonne, 2019	Yes	Sampling a large amount of water and WGS have been decisive for the root cause analysis of the infection. Our findings prove, if necessary, the usefulness of WGS for typing purposes in the M. chelonae species.
Gu, 2020	Yes	We show that all M. chelonae isolates tested were highly dissimilar, as indicated by high pairwise SNV values, consistent with environmental acquisition and not a nosocomial point source.
Labuda, 2020	Yes	For phylogenetic analyses, 13 isolates were nearly identical. Isolate 14 was also highly related. Together, these data provide evidence of a common source.
Doyle, 2020	Yes	We found WGS to be useful to confirm whether different patients' strains are unrelated, even within the dominant clones, but it has

Publication First Author, Year	Did sequencing aid in identifying point source of outbreak?	Author comments
		been far more difficult to reach definite conclusions about cross-transmission
Ghodousi, 2020	Yes	Finally, we shown that WGS is the preferred method to distinguish whether a clinical strain is related to the HCU outbreak strain.
Wetzstein, 2020	Yes	We found no evidence for nosocomial patient-to-patient transmissions, although patients with closely related strains and treated at the same center were identified.
Xu, 2021	Yes	The WGS of 11 available M. chimaera patient isolates and 13 available M. chimaera environmental isolates indicated that all were closely related to each other and to previous isolates from the international outbreak identified in 2015.
Lipworth, 2021	Yes	Over the past 6 years, Public Health England (PHE) has implemented whole-genome sequencing (WGS) to replace existing reference laboratory techniques for all mycobacteria, producing a near-complete dataset of all sequenced *M. abscessus* clinical isolates in England from patients with and without cystic fibrosis. These data have been linked to routinely collected health-care usage datasets, presenting an opportunity to investigate person-to-person transmission of *M. abscessus* on a national scale across all patient groups.
Lecorche, 2021	Yes	We found very low diversity in the epidemic cluster for the M. chimaera isolates in France, with a mean pairwise distance of 4 SNPs, and confirmed that the 2 suspected patients were infected by the M. chimaera epidemic strain.
Gross, 2022	Yes	Using integrated analysis of epidemiologic investigations, healthcare environmental sampling, mapping home of residence watershed, and determination of strength of cluster relatedness with pan-genome analysis, this study suggests healthcare-associated transmission of NTM is uncommon in our center.
Fujiwara, 2022	Not reported	
Waglechner, 2022	No	We show WGS data is necessary but not sufficient to establish patient-to-patient M. abscessus transmission
Daniau, 2022	Yes	WGS appears to be a suitable tool for the molecular investigation of NTM infections, but might need expert rules and standardization to be used further.
Inkster, 2022	Yes	WGS is a promising adjunct for the investigation of outbreaks due to atypical mycobacteria.
Davidson, 2022	Yes	Prompt and thorough investigation of HCFA *M. abscessus* clusters is essential for mitigation of potential outbreaks and prevention of new infections. Molecular techniques, including WGS, have become critical components of such investigations; however, despite its power, WGS of NTM isolates has limitations.

**Publication First Author, Year**	**Did sequencing aid in identifying point source of outbreak?**	**Author comments**
Gross, 2023	Yes	However, this investigation does support healthcare-associated M. chimaera acquisition on the basis of a genetic match with a hospital water fountain isolate.
Dedrick, 2023	Not reported	
Nagano, 2023	Yes	In conclusion, we used the WGS analysis to genetically demonstrate a pseudo-outbreak of *M. lentiflavum* in our hospital.
Komiya, 2023	Yes	Whole-genome sequencing revealed a potential nosocomial outbreak among patients who were ventilator dependent and without CF.
Faury, 2023	Yes	Whole-genome sequencing showed that the same strain was isolated from the shared shower water of the unit.
Kling, 2023	Yes	Our findings suggest that isolation of *M. xenopi* from pulmonary specimens may be increasing, further defines the genomic population structure of this potentially emerging infection and establishes WGS as a useful tool for outbreak investigation strain typing.
Groenewold, 2023	Yes	We conducted environmental sampling of the HCW's home and identified an outdoor hot tub at the HCW's home as the possible source of *M. wolinskyi.* These results were confirmed by PFGE and WGS analyses comparing clinical and environmental mycobacterial isolates.
Klompas, 2023	Yes	Whole-genome sequencing confirmed the presence of a genetically identical element in ice and water machine and patient specimens.

Supplemental Table 6. Genomic methods used as part of NTM transmission investigation

Publication First Author, Year	WGS (Yes/No)	Accessory Genome (Yes/No)	Signature SNP (Yes/No)
Bryant, 2013	Yes	No	No
Harris, 2015	Yes	No	No
Robinson, 2016	Yes	No	No
Chand, 2016	Yes	No	No
Everall, 2017	Yes	No	No
Yan, 2020	Yes	No	No
Hasan, 2019	Yes	No	No
Decalonne, 2019	Yes	No	No
Gu, 2020	Yes	Yes	No
Labuda, 2020	Yes	No	No
Doyle, 2020	Yes	Yes	No
Ghodousi, 2020	Yes	No	Yes
Wetzstein, 2020	Yes	No	No
Xu, 2021	Yes	No	No
Lipworth, 2021	Yes	No	No
Lecorche, 2021	Yes	No	No
Gross, 2022	Yes	Yes	No
Fujiwara, 2022	Yes	No	No
Waglechner, 2022	Yes	No	No
Daniau, 2022	Yes	No	No
Inkster, 2022	Yes	No	No
Davidson, 2022	Yes	Yes	No
Gross, 2023	Yes	Yes	No
Dedrick, 2023	Yes	No	No
Nagano, 2023	Yes	Yes	No
Komiya, 2023	Yes	Yes	Yes
Faury, 2023	Yes	No	No
Kling, 2023	Yes	No	No
Groenewold, 2023	Yes	No	No
Klompas, 2023	Yes	No	No

Supplemental Table 7. Genomic analysis performed by study authors in addition to SNP analysis

**Publication First Author, Year**	**Additional genomic analysis**
Bryant, 2013	Mutation Rate
Harris, 2015	Multilocus sequence typing<br>Antibiotic resistance
Robinson, 2016	None
Chand, 2016	Phylogenetic analysis
Everall, 2017	Phylogenetic analysis
Yan, 2020	None
Hasan, 2019	Phylogenetic analysis
Decalonne, 2019	None
Gu, 2020	Resistance gene identification
Labuda, 2020	None
Doyle, 2020	Multilocus sequence typing<br>Phylogenetic analysis
Ghodousi, 2020	Phylogenetic analysis
Wetzstein, 2020	None
Xu, 2021	None
Lipworth, 2021	Phylogenetic analysis
Lecorche, 2021	Phylogenetic analysis
Gross, 2022	Phylogenetic analysis
Fujiwara, 2022	Phylogenetic analysis
Waglechner, 2022	No
Daniau, 2022	Yes
Inkster, 2022	None
Davidson, 2022	Phylogenetic analysis<br>Pangenome analysis
Gross, 2023	Phylogenetic analysis<br>Pangenome analysis
Dedrick, 2023	Dominant circulating clones<br>Prophage identification<br>Phylogenetic analysis
Nagano, 2023	Multilocus sequence typing
Komiya, 2023	Phylogenetic analysis
Faury, 2023	None
Kling, 2023	Phylogenetic analysis
Groenewold, 2023	Pulsed-field gel electrophoresis
Klompas, 2023	None

Supplemental Table 4. Anatomical sites of non-tuberculous mycobacterial isolates from cluster investigations aided by whole genome sequencing

**Anatomic Site**	Pulmonary			Skin/Soft Tissue			Blood			Bone		
**Publication First Author, Year**	Total Sequenced Study Case Isolates	Sequenced Isolates - Transmitted	Sequenced Isolates - Not Transmitted	Total Sequenced Study Case Isolates	Sequenced Isolates - Transmitted	Sequenced Isolates - Not Transmitted	Total Sequenced Study Case Isolates	Sequenced Isolates - Transmitted	Sequenced Isolates - Not Transmitted	Total Sequenced Study Case Isolates	Sequenced Isolates - Transmitted	Sequenced Isolates - Not Transmitted
Bryant, 2013	31	17	14									
Harris, 2015	27	0	27									
Robinson, 2016	1	1	0									
Everall, 2017				188	188	0						
Decalonne, 2019				1	1	0						
Yan, 2020	17	7	10									
Gu, 2020	3	0	3	4	0	4						
Labuda, 2020							14	14	0			
Doyle, 2020	134	0	134				1	0	1			
Ghodousi, 2020	1	1	0				19	19	0			
Wetzstein, 2020	38	0	38									
Lecorche, 2021							2	2	0	1	1	0
Waglechner, 2022	169	7	162									
Gross, 2022	80	5	75									
Inkster, 2022				1	1	0	1	1	0			
Davidson, 2022	4	4	0	2	2	0	1	1	0			
Gross, 2023	23	8	15									
Wetzstein, 2022	154	0	154									
Nagano, 2023	22	18	4									
Komiya, 2023	52	52	0									
Faury, 2023							1	1	0			
Kling, 2023	6	0	6									
Groenewold, 2023										8	8	0
Klompas, 2023				2	2	0	2	2	0			
**Total sequenced isolates**	762	120	642	198	194	4	41	40	1	9	9	0
**% of all sequenced isolates by anatomic site**	74.3			19.3			4.0			0.9		
**% of sequenced isolates implicated in transmission by anatomic site**		15.7			98.0			97.6			100.0	
**% of all implicated sequenced isolates by anatomic site**		32.8			53.0			10.9			2.5	

**Anatomic Site**	Gastrointestinal/Intraabdominal			Lymph node			Tissue			CNS		
**Publication First Author, Year**	Total Sequenced Study Case Isolates	Sequenced Isolates - Transmitted	Sequenced Isolates - Not Transmitted	Total Sequenced Study Case Isolates	Sequenced Isolates - Transmitted	Sequenced Isolates - Not Transmitted	Total Sequenced Study Case Isolates	Sequenced Isolates - Transmitted	Sequenced Isolates - Not Transmitted	Total Sequenced Study Case Isolates	Sequenced Isolates - Transmitted	Sequenced Isolates - Not Transmitted
Bryant, 2013												
Harris, 2015												
Robinson, 2016												
Everall, 2017												
Decalonne, 2019												
Yan, 2020												
Gu, 2020												
Labuda, 2020												
Doyle, 2020	11	0	11									
Ghodousi, 2020							2	2	0	1	1	0
Wetzstein, 2020				1	0	1						
Lecorche, 2021												
Waglechner, 2022												
Gross, 2022												
Inkster, 2022												
Davidson, 2022												
Gross, 2023												
Wetzstein, 2022												
Nagano, 2023												
Komiya, 2023												
Faury, 2023												
Kling, 2023												
Groenewold, 2023												
Klompas, 2023												
**Total sequenced isolates**	11	0	11	1	0	1	2	2	0	1	1	0
**% of all sequenced isolates by anatomic site**	1.1			0.1			0.2			0.1		
**% of sequenced isolates implicated in transmission by anatomic site**		0.0			0.0			100.0			100.0	
**% of all implicated sequenced isolates by anatomic site**		0.0			0.0			0.5			0.3	

Note: A publication not listed indicates the data was not available to report
